# Melt Rheological Behavior and Morphology of Poly(ethylene oxide)/Natural Rubber-*graft*-Poly(methyl methacrylate) Blends

**DOI:** 10.3390/polym12030724

**Published:** 2020-03-24

**Authors:** Nurul Fatahah Asyqin Zainal, Say Aik Lai, Chin Han Chan

**Affiliations:** 1 Centre of Foundation Studies, Universiti Teknologi MARA, Cawangan Selangor, Kampus Dengkil, Dengkil 43800, Selangor, Malaysia; nurulfatahah@gmail.com; 2 TA Instruments Sdn. Bhd. D7-1-G Block D7 Pusat Perdagangan Dana 1, Jalan PJU 1A/46, Petaling Jaya 47301, Selangor, Malaysia; JLai@tainstruments.com; 3 Faculty of Applied Sciences, Universiti Teknologi MARA, Shah Alam 40450, Selangor, Malaysia

**Keywords:** graft copolymer, elasticity, viscosity, droplet–matrix, co-continuous

## Abstract

The influence of morphology on the rheological properties of poly(ethylene oxide) (PEO) and natural rubber-*graft*-poly(methyl methacrylate) (NR-*g*-PMMA) blends in the melt was investigated. The blends were prepared at different blend compositions by a solution-casting method. Linear viscoelastic shear oscillations measurements were performed in order to determine the elastic and viscous properties of the blends in the melt. The rheological results suggested that the blending of the two constituents reduced the elasticity and viscosity of the blends. The addition of an even small amount of NR-*g*-PMMA to PEO changed the liquid-like behavior of PEO to more solid-like behavior. Morphological investigations were carried out by optical microscopy to establish the relationship between morphology and melt viscosity. Depending on the blend compositions and viscosities, either droplet–matrix or co-continuous morphologies was observed. PEO/NR-*g*-PMMA blends exhibited a broad co-continuity range, and phase inversion was suggested to occur at the PEO/NR-*g*-PMMA blend with a mass ratio of 60/40 (*m*/*m*), when NR-*g*-PMMA was added to PEO as a matrix.

## 1. Introduction

Polymer blends have drawn marked attention over years in producing suitable materials by mixing polymers for specific applications, which may be cheaper and provide less time of production than the development of new monomers or new polymerization routes [1,2]. Polymer blends are also widely used as polymer electrolytes, and some have proven to be promising candidates in solid polymer electrolytes (SPEs) [3,4] for the application of rechargeable batteries. One of the widely used polymer hosts in SPEs is poly(ethylene oxide) (PEO) due to a large variety of inorganic salts that are soluble in PEO [5,6] in the molten state or in the amorphous phase of high-molar-mass PEO at room temperature. However, high-molar-mass PEO (*M*_η_ ≥ 300,000 g·mol^−1^) with lithium salt systems exhibits low conductivity (~10^−5^ S·cm^−1^) at room temperature [4,7,8,9,10]. Adding a second component to high-molar-mass PEO (i.e., polymer blending) may improve the ionic conductivity of systems in comparison to those of PEO/salt systems at room temperature [4,11,12,13,14]. Besides, the enhancement of mechanical properties of polymer/salt blends may be achieved by blending them with other polymers [13,15,16] without sacrificing the ionic conductivity of the composites. Hence, it is of vital importance to have more insight on PEO-based binary blends before the elucidation of the properties of the ternary systems of PEO-based blends with the addition of inorganic salt.

High-molar-mass polymers give rise to a limited entropy gain upon blending. Hence, most binary polymer blends are immiscible [17], of which the system is with coexisting phases each being pure or very rich in one of the constituents. Generally, immiscible blends give rise to different morphologies, such as droplet-matrix morphology [18,19] and co-continuous morphology [20,21,22,23], which influence the properties of blends and govern the performance of systems. For instance, a fibrillar morphology of polypropylene (PP)/polyethylene (PE) enhances the tensile properties of PE [24], a droplet-matrix morphology of the random copolymer of polypropylene with ethylene (PP-R)/poly(ethylene-octene) (POE) blends improves the impact properties of POE [25] and a co-continuous morphology, where both phases percolate in polystyrene (PS)/polybutadiene (PB) blends, increases the tensile strength of PB [26]. 

Morphologies in immiscible binary blends lead to a complex rheological behavior [27,28,29], particularly in a low-frequency region, since the domain sizes of different components in systems are close to the micrometer scale. Few studies reported on the morphologies and rheological properties of PEO-based blends in the molten state. For instance, a work from [30] studied the viscoelasticity of PEO (*M_w_* = 120,000 g·mol^−1^; glass transition temperature *T*_g_ = −55 °C; melting temperature *T*_m_ = 70 °C) and poly(vinylidenefluoride-co-hexafluoropropylene) P(VDF-co-HFP) (*M_w_* = 115,000 g·mol^−1^; *T*_g_ = −29 °C; *T*_m_ = 135 °C) in a low-frequency region. The representation of storage modulus ($G^{\prime }$) as a function of blend composition at *ω* = 0.01 rad·s^−1^ showed the elasticity of the blends was higher as compared to that of the parent polymers. The influence of the morphologies of the blends on $G^{\prime }$ in the low-frequency region can be observed [30]. Another work [31] investigated the existence of a co-continuous morphology of PEO (*M_w_* = 400,000 g·mol^−1^)/PS (*M_w_* = 150,000 g·mol^−1^) blends by using a dynamic stress rheometer. Data of $G^{\prime }$ for all PEO/PS blends taken at *ω* = 0.01 and 0.1 rad·s^−1^ were plotted as a function of PEO content. Two maxima of $G^{\prime }$ were noted at the blends containing 40 and 70 wt % of PEO and were correlated to the co-continuous morphology for the blends in a composition range of 40–70 wt % of PEO in the PEO/PS blends.

There are not many rheological studies on blending of PEO with a copolymer containing hard and soft segments [32,33]. Blends of PEO with natural rubber-graft-poly(methyl methacrylate) polymer (NR-*g*-PMMA) (mole ratio of NR and PMMA-graft polymer: 60%:40%), where the NR backbone is the soft segment and the PMMA-graft polymer is the hard segment, may serve as interesting polymer hosts for SPEs. The PEO phase will be for the ion percolation [34,35,36], while the hard segment of the PMMA-graft polymer (with higher *T*_g_) is for the mechanical strength and the NR backbone (with lower *T*_g_) is for the impact resistance. A relatively good damping property of NR-*g*-PMMA was also reported, which was described by the broadening of the tanδ peak (or $G^{\prime \prime }$/$G^{\prime }$) [37]. The properties of ternary systems of PEO/NR-*g*-PMMA with the addition of inorganic salt will be discussed in a forthcoming publication. The thermal properties and the morphologies at 25 °C of these binary blends were reported in [38,39].

In this work, rheological studies in a low-frequency region at 140 °C (above *T*_m_ of PEO) were used to evaluate the polymer chain relaxation behavior in correlation to the morphologies of PEO/NR-*g*-PMMA binary blends. We carried out oscillatory experiments within a linear viscoelastic (LVE) region, where the linear relationship between the applied force and the measured quantities can be observed. In this study, we focused on the measured quantities as a function of frequency at 140 °C. Subsequently, a phenomenological approach was utilized to evaluate the dynamics of neat polymers and the blends. Optical microscopy (OM) was used to assess the morphologies of the blends in the melt. Regulating the morphologies of the blends can easily be achieved by changing the blend composition, which in turns governs the rheological behavior of the systems. Understanding of the polymer chain relaxation behavior in the molten state is important for the blend processability for commercial application.

## 2. Materials and Method

### 2.1. Materials and Sample Preparation

Both polymers of PEO and NR-*g*-PMMA used in this work were purified before the blend preparation. For purification, PEO was dissolved in chloroform (CHCl_3_) (Merck, Darmstadt, Germany) and precipitated in *n*-hexane (Merck, Darmstadt, Germany). NR-*g*-PMMA was purified through the removal of macrogel with simple filtration using a nylon filter with an approximately 1-μm pore size and the removal of homopolymers using the selective extraction of homopolymers from the graft copolymer. The free NR homopolymer (ungrafted NR) and the free PMMA homopolymer were extracted with light petroleum ether (Merck, Darmstadt, Germany) and acetone (Merck, Darmstadt, Germany), respectively, at 40–50 °C for 24 h. The remaining product, which consisted of microgels, was dried to a constant weight at 80 °C. The characteristics of the purified PEO and NR-*g*-PMMA used in this work are tabulated in Table 1. As for the removal of the microgels in the NR, ultracentrifugation followed by filtration with a 0.45-μm pore size may be possible. However, this procedure is not viable for commercial application. Hence, the removal of microgels was not attempted in this study.

For the blends preparation, a solution-casting technique was used to prepare free standing PEO/NR-*g*-PMMA films. These films were prepared from solutions of these two polymers with a mass ratio of 2%:98% in tetrahydrofuran (THF) (Merck, Darmstadt, Germany). The solution was stirred for 24 h at 50 °C, until the polymers were completely dissolved. Next, the homogeneous solutions were poured into Teflon dishes and dried under a fume hood for a few days at room temperature, before they were dried at 50 °C for 24 h in an oven. The films were dried under nitrogen atmosphere at 80 °C for 30 min before isothermally crystallized at 25 °C for 24 h. The films were vacuum-dried at 25 °C for 24 h and were kept in dessiccators at 25 °C. The mass ratios (*m*/*m*) of the PEO/NR-*g*-PMMA blends prepared in this study were 100/0, 90/10, 70/30, 60/40, 50/50, 40/60, 10/90, and 0/100.

### 2.2. Rheological Measurements

Rheological analysis on blend samples was performed using a Discovery Hybrid Rheometer (DHR3), (TA Instruments, New Castle, DE, USA) and was operated using Trios software (TA Instruments, New Castle, DE, USA). A 20-mm parallel-plate geometry was used, and the gap between the plate and the sample was fixed at 0.5 mm in order to ensure the normal force control and good contact of the sample with the plate. For the selection of an appropriate strain value within an LVE range, both the neat PEO and NR-*g*-PMMA were subjected to strain sweeps ranging from 0.001% to 10% strain at a 1.9 Hz with 20 points per decade. This LVE range was subsequently used in frequency sweep experiments. Next, frequency sweeps ranging from 0.01 to 100 Hz at a 0.1% strain with 20 points per decade were performed at 140 °C to study the rheological behavior of the samples in the molten state. The temperature was controlled using a Peltier plate controller unit and a Thermocube waterbath (Princeton Instruments, Trenton, NJ, USA). The time for annealing the samples was 5 min before each measurement. The slopes of $G^{\prime }$ and $G^{\prime \prime }$ in the low-frequency region were calculated based on a linear regression function. We noted that each regression function referred to one rheological measurement. Hence, we estimated the error of the result of the linear regression based on a 2-tailed student *t*-test distribution with a confidence level of 95%. The complex modulus (|G*|) can be represented by:

(1) $$\left|G*\right|(\omega )=G^{\prime }(\omega )+iG^{\prime \prime }(\omega ).$$

According to the Maxwell model theory of linear viscoelasticity, $G^{\prime }$ and $G^{\prime \prime }$ can be written as:

(2) $$G^{\prime }=G\frac{{\left(\tau \omega \right)}^{2}}{\left(1+\omega^{2}\tau^{2}\right)},\;G^{\prime \prime }=G\frac{\tau \omega }{\left(1+\omega^{2}\tau^{2}\right)},$$

where $G^{\prime }$ denotes the storage modulus (or elasticity or reversible deformation), $G^{\prime \prime }$ denotes the loss modulus (or flow or nonreversible deformation), ω is angular frequency and τ is the relaxation time. At a sufficiently low frequency (ω→0), $G^{\prime }$ and $G^{\prime \prime }$ in Equation (2) were rewritten as:

(3) $$G^{\prime }=G{\left(\omega \tau \right)}^{2},\;G^{\prime \prime }=G(\omega \tau ),\;G^{\prime }\propto \omega^{2},\;G^{\prime \prime }\propto \omega .$$

This means that, in a double logarithmic plot in a low-frequency region, the slopes of $G^{\prime }$ and $G^{\prime \prime }$ versus ω curves can be expressed by power law exponents of 2 and 1, respectively.

The fundamental relationship between quantities G and viscosity (η) was given as:

(4) $$\eta^{\prime }(\omega )=\frac{G^{\prime \prime }(\omega )}{\omega },\;\eta^{\prime \prime }(\omega )=\frac{G^{\prime }(\omega )}{\omega },$$

where $\eta^{\prime }$ and $\eta^{\prime \prime }$ represent the real and imaginary parts of complex viscosity (|η*|), respectively, which is a measure of the total resistance of a material to flow as a function of ω and defined as:

(5) $$\left|\eta *\right|(\omega )=\eta^{\prime }(\omega )+i\eta^{\prime \prime }(\omega ).$$

### 2.3. OM

A Soptop CX40M polarizing microscope (Sunny Optical Technology, Ningbo, China) equipped with a magnification lense with a magnification of 10× *g* and a Linkam TM 600/s hotstage (Surrey, England, UK) along with iSolution software (Lite x64, Easley, SC, USA) was used to study the morphologies of the blends. A sample with a *m*/*v* ratio of 1% in THF was heated overnight at 50 °C. Upon complete dissolution, the polymer solution was placed drop-wise on top of a glass cover slip, allowed to dry at room temperature and further dried in a vacuum oven at 25 °C for 24 h. 

The dried sample was heated from room temperature to 140 °C at a rate of 10 °C·min^−1^. The sample was annealed at 140 °C for 10 min. Micrographs were captured at 140 °C at a 1 min interval, and more than 5 micrographs were captured at different spots to obtain the representative sample at a 10× *g* magnification.

## 3. Results and Discussion

### 3.1. Rheological Properties of the Parent Polymers

**Figure 1:**
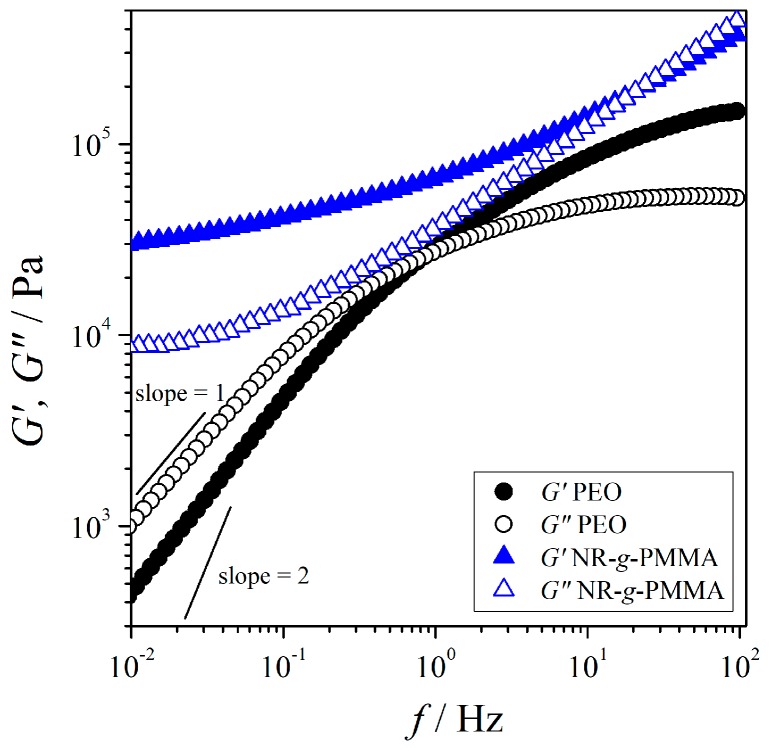
The variation of $G^{\prime }$ (solid markers) and $G^{\prime \prime }$ (open markers) with frequency at *T* = 140 °C. The results for PEO are indicated by circles; the results for NR-*g*-PMMA are indicated by triangles.

The variations of $G^{\prime }$ and $G^{\prime \prime }$ for both neat PEO and NR-*g*-PMMA as a function of frequency are shown in Figure 1. Based on the Maxwell model (*cf*. Equation (3)), a polymer melt is fully relaxed (i.e., one relaxation time), when $G^{\prime }$ and $G^{\prime \prime }$ obey the power law dependence in a low-frequency region with the slopes equal to 2 and 1, respectively. The linear regression on the double logarithmic plots of $G^{\prime }$ and $G^{\prime \prime }$ moduli versus frequency allows for the estimation of power law exponents for $G^{\prime }$ and $G^{\prime \prime }$, which can be extracted from the slopes of the regression curves using Equation (3) in the low-frequency region. As for the neat PEO, the estimated power law exponents (refer to Figure 1 and Table 2) showed that only the viscous part was almost relaxed whereas the elastic part did not return to equilibrium (or not fully relax). Since the viscoelastic properties in the low-frequency region reflected the long-range motion of polymer chains, this deviation may indicate the distribution of relaxation times of PEO chains in the molten state. This observation is in agreement with light scattering studies reported by Walter et al. [6] and Walkenhorst et al. [41], where they observed the PEO melt was not uniform on the microscopic scale due to the structured random network formed by extensive inter- and intrapolymer connections. A rheological study on PEO (*M_w_* = 300,000 g·mol^−1^; *T*_g_ = −57 °C) measured at *T* = 80 °C also reported deviations in both $G^{\prime }$ and $G^{\prime \prime }$ from terminal viscoelastic relaxation, which indicated the restrictions of the long-range motion of PEO (data are listed in Table 2) [42]. Furthermore, the heterogeneities of PEO can also be observed using impedance spectroscopy at 25 °C by adding very low salt concentrations (e.g., 0.05 wt %) due to the formation of a percolation network of conductive (polymer-poor) and dielectric (polymer-rich) domains [10].

$G^{\prime \prime }$ did not exhibit a maximum under the experimental condition, where the maximum of $G^{\prime \prime }$ was expected at *f* > 10^2^ Hz. The crossing of the $G^{\prime }$ and $G^{\prime \prime }$ functions $\left(f_{\mathrm{cross}}^{G^{\prime },G^{\prime \prime }}\right)$ appeared at a frequency below the frequency of the maximum of $G^{\prime \prime }$, $\left(f_{\max}^{G^{\prime \prime }}\right)$. $\left(f_{\mathrm{cross}}^{G^{\prime },G^{\prime \prime }}\right)$ < $\left(f_{\max}^{G^{\prime \prime }}\right)$ implied the dispersion of the relaxation times of PEO chains in the melt. In addition, $G^{\prime \prime }>G^{\prime }$ was observed at low frequencies, which suggested that PEO behaved liquid-like at *T* = 140 °C.

The $G^{\prime }$ and $G^{\prime \prime }$ moduli of neat NR-*g*-PMMA depended weakly on frequency in the low-frequency region, as shown in Figure 1 and Table 2. Both $G^{\prime }$ and $G^{\prime \prime }$ of NR-*g*-PMMA were far away from the terminal relaxation, and large-scale relaxations were effectively restrained in NR-*g*-PMMA. This behavior may be attributed to the network of crosslinked chains (e.g., microgels), which restricted the long-range motion of chains. Works have been reported that the extracted NR contained nitrogen-containing compounds even after extensive purification [43,44]. These nitrogeneous compounds, namely proteins and phospholipids, formed an intermolecular interaction at the NR chain ends via hydrogen bonding, which possibly formed branching and gels [43,45,46], as a naturally occurring network [47,48]. This is in agreement with the correlation between the protein content and the gel content [49], which suggested that proteins were involved in the majority of crosslinks in NR [45,50]. Furthermore, these proteins were also suggested to be involved in the structuring of microaggregates, also called as “microgel” [44]. Of course, we cannot rule out completely the PMMA graft polymer may also lead to the restriction of the large-scale relaxation of NR-*g*-PMMA. The relatively high values of $G^{\prime }$ and $G^{\prime \prime }$ of NR-*g*-PMMA at low frequencies may imply that the microgel was still present in the NR-*g*-PMMA sample after purification using a 1 μm-pore-size nylon filter. Contrary to PEO, NR-*g*-PMMA behaved like an elastic solid, since $G^{\prime }>G^{\prime \prime }$ was observed at low frequencies at *T* = 140 °C.

**Figure 2:**
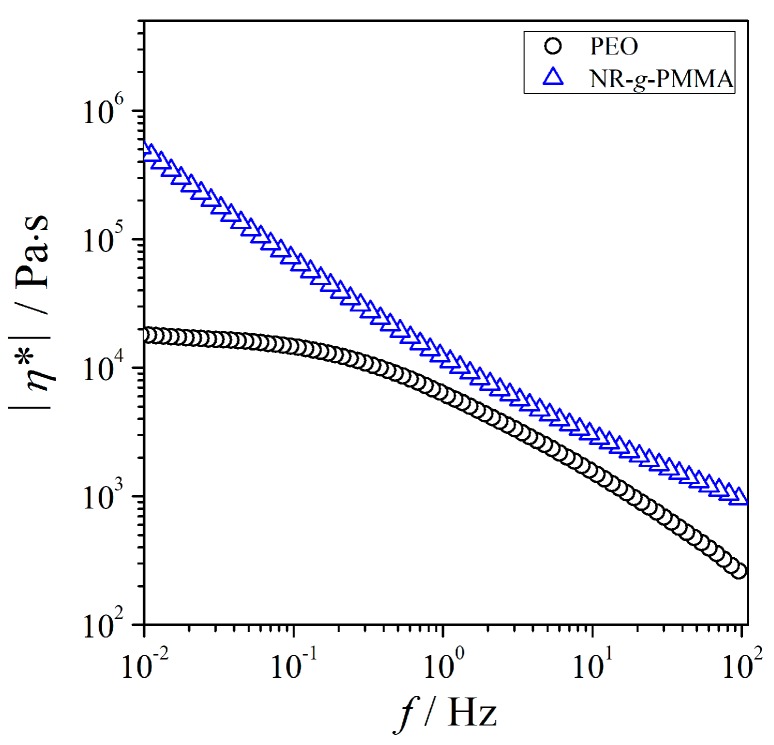
Plots of complex viscosity as a function of frequency for PEO and NR-*g*-PMMA at *T* = 140 °C. The results for PEO are indicated by circles; the results for NR-*g*-PMMA are indicated by triangles.

Figure 2 presents the plots of |η*| as a function of frequency for both the neat PEO and NR-*g*-PMMA at *T* = 140 °C. For the frequency range studied, the |η*| values of NR-*g*-PMMA were considerably greater than those of PEO. The |η*| value of PEO showed relatively constant, behaved as a Newtonian fluid at frequencies below 0.1 Hz and decreased as the frequency increased. Conversely, the |η*| values of NR-*g*-PMMA did not reach a constant value within the experimental frequency range but continued to increase with the decreasing frequency. In other words, NR-*g*-PMMA showed a shear-thinning behavior throughout the frequency under discussion. Additionally, the high viscosity of NR-*g*-PMMA at *T* = 140 °C indicated the flow restrictions in the chains, which restricted the long-range motion of the chains as described earlier in the modulus results. The difference between the viscosities of NR-*g*-PMMA and PEO also increased as the frequency decreased. 

### 3.2. Rheological Characterization of the PEO/NR-g-PMMA Blends

#### 3.2.1. Variation of Viscoelastic Modulus Function with Blend Composition

**Figure 3:**
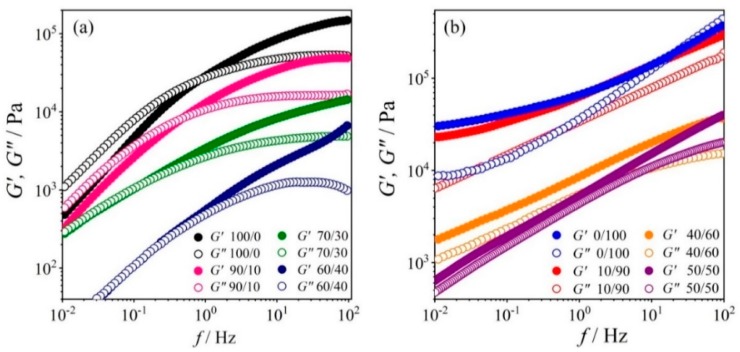
Plots of storage modulus (closed marker) and loss modulus (open marker) as a function of frequency for PEO/NR-*g*-PMMA at *T* = 140 °C: (**a**) blends with the excess PEO content and (**b**) blends with the excess NR-*g*-PMMA content.

Figure 3a,b presents the variations of $G^{\prime }$ and $G^{\prime \prime }$ with frequency for PEO/NR-*g*-PMMA blends with excess of PEO and excess of NR-*g*-PMMA, respectively. The power law exponents by linear regression according to Equation (3) are listed in Table 2. The behavior of the blends in the low-frequency region showed that the large-scale polymer chain relaxations were restrained with the ascending NR-*g*-PMMA content in the blends. The power law exponents for both $G^{\prime }$ and $G^{\prime \prime }$ decreased with the increasing content of NR-*g*-PMMA in the blends, which implied that both the elastic and viscous parts of |G*| did not return to equilibrium (i.e., the broad distribution of relaxation times). The addition of a minor amount of NR-*g*-PMMA to PEO did change the terminal behavior of PEO in the low-frequency region. PEO became more solid-like in the low- as well as high-frequency ranges even after the addition of only 10 wt % of NR-*g*-PMMA. The transition from liquid-like to solid-like behavior appeared at very low concentrations of NR-*g*-PMMA in the blends, as shown in Figure 3a.

The plateau-like modulus of $G^{\prime }$, *G*°, of the neat PEO appeared at above 10^5^ Pa. Upon the addition of the mass fraction of NR-*g*-PMMA (*W*_NR-*g*-PMMA_) of 0.1 to PEO, the *G*° value declined to 4.9 × 10^4^ Pa and further decreased when *W*_NR-*g*-PMMA_ of 0.4 was added to PEO. The plateau region of $G^{\prime }$ originated from thermoreversible crosslinks like the entanglements of the chains in the melt. The frequency of the liquid-to-solid transition shifted to lower values for these blends. Consequently, *G*° and $f_{\mathrm{cross}}^{G^{\prime },G^{\prime \prime }}$ shifted to lower values with the increasing content of NR-*g*-PMMA added to PEO. In other words, the addition of NR-*g*-PMMA to PEO led to the transition from liquid-like to solid-like behaviors of the blend. This behavior was also observed in a blend system comprising natural rubber-*graft*-poly(butyl acrylate) (NR-*g*-PBA) and polylactide (PLA), where NR-*g*-PBA toughened the PLA [51]. 

On the other side, the addition of PEO to NR-*g*-PMMA did not change the solid-like behavior of NR-*g*-PMMA (*cf.* Figure 3b). The storage modulus of the blend decreased with the increasing content of PEO. In addition, the difference between $G^{\prime }$ and $G^{\prime \prime }$ in the low-frequency region decreased with the ascending PEO content.

In summary, blends with less than 60 wt % of PEO content behaved solid-like at *T* = 140 °C, and the long-range motions of polymer chains were restricted. The distance between $G^{\prime }$ and $G^{\prime \prime }$ diminished in 60/40 and 50/50 blends with co-continuous morphology at low frequencies (refer to Section 3.3). Hence, the phase inversion was suggested to appear around these blend compositions. For *W*_PEO_ ≥ 0.7, PEO formed the matrix, and NR-*g*-PMMA was the dispersed phase; for *W*_NR-*g*-PMMA_ ≥ 0.7, NR-*g*-PMMA formed the matrix, and PEO turned into the dispersed phase (refer to Section 3.3). This morphology remained in the blends with higher contents of NR-*g*-PMMA, where these blends behaved as solid-like without transition to liquid-like behavior for the studied frequency range.

**Figure 4:**
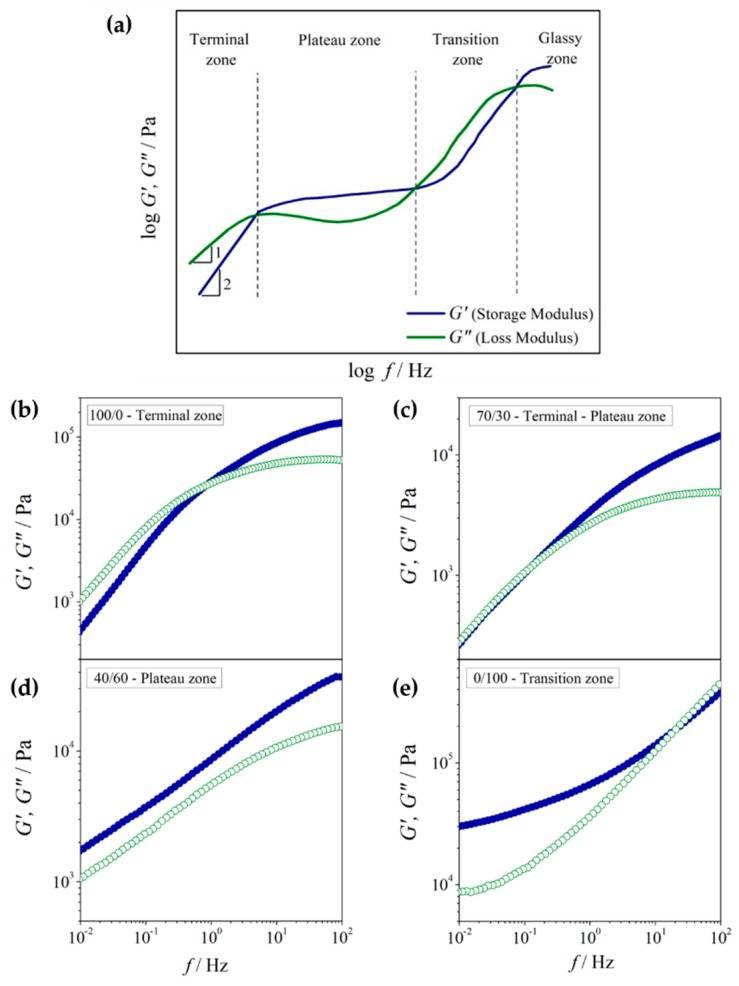
Plots of $G^{\prime }$ and $G^{\prime \prime }$ as a function of frequency for (**a**) three-zone model and PEO/NR-*g*-PMMA blends of (**b**) 100/0 (**c**) 70/30 (**d**) 40/60 and (**e**) 0/100.

A three-zone model [52,53], as shown in Figure 4a, was referred to in order to further interpret the modulus curves of the PEO/NR-*g*-PMMA blends. Based on the relative values of $G^{\prime }$ and $G^{\prime \prime }$, a logarithmic plot of moduli consisted of terminal, plateau, and transition zones. The time-scale associated with the terminal zone corresponded to long-range relaxations, where the polymer chain relaxation happened faster than the deformation and the chains showed translational diffusion. Conversely, the time-scale of the transition zone was related to short-range relaxations, which involved the local segmental relaxation occurring in high-frequency regions [53]. Referring to the moduli curves in Figure 4b,e, PEO showed a terminal–plateau–zone behavior, whereas NR-*g*-PMMA exhibited a plateau-transition zone. This indicated that NR-*g*-PMMA had a longer relaxation time than PEO, as its terminal-zone behavior would be expected to appear in a very-low-frequency region, which was beyond the accessible frequencies of the instrument. As for the blends, $f_{\mathrm{cross}}^{G\prime ,G^{\prime \prime }}$ shifted to the low-frequency region, as the NR-*g*-PMMA content increased. This described the shifting of the terminal-zone behavior to low frequencies with an increase in NR-*g*-PMMA content in the blends, as presented in Figure 4b–e. 

Furthermore, the moduli curves in Figure 4a represented a typical entangled homopolymer [54] or a single-phase blend system [55], where both moduli followed $G^{\prime }\propto \omega^{2}$ and $G^{\prime \prime }\propto \omega$ in the low-frequency region as described in Equation (3). The deviation from this LVE theory indicated phase separation in the polymer blends [56,57]. As presented in Figure 4b–e, both the power law exponents of $G^{\prime }$ and $G^{\prime \prime }$ decreased with the addition of NR-*g*-PMMA to PEO, which indicated phase separation behavior in the PEO/NR-*g*-PMMA blends. Similar phenomena were also observed for these phase-separated blends [58,59], where the deviation of LVE occurred in low-frequency regions.

#### 3.2.2. Variation of Melt Viscosity as a Function of Blend Composition

**Figure 5:**
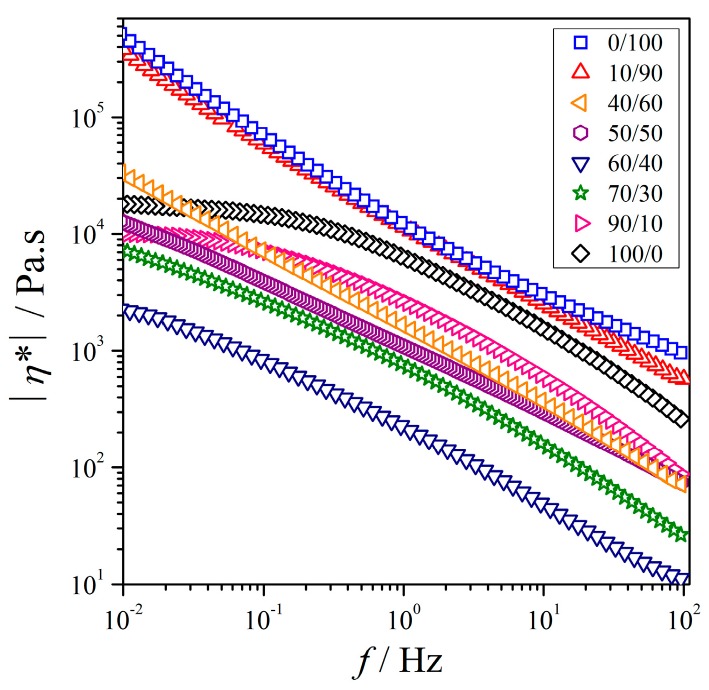
Complex viscosity versus frequency for PEO/NR-*g*-PMMA blends at *T* = 140 °C.

The |η*| values of the PEO/NR-*g*-PMMA blends are shown in Figure 5. For the neat PEO and the blend with a mass ratios (*m*/*m*) of 90/10, |η*| was independent of frequency at low frequencies, which corresponded to the Newtonian zone. When *W*_NR-*g*-PMMA_ > 0.10 was added to PEO, |η*| became dependent on frequency for the entire experimental frequency range. It implied that these blends showed shear thinning behavior and long-range motion restrictions in the polymer chains at *T* = 140 °C.

Next, the viscosity properties of the blends in the low-frequency region were discussed qualitatively. We looked at the minimum frequency of |η*|. For this qualitative evaluation, *f* = *const* at a sufficiently low frequency was used. At lower frequencies, the polymer chains had sufficient time to free themselves from the chain entanglement, and hence chain relaxations were fully developed. The evaluation in the low-frequency region of the viscosity of the blends correlated to the morphological properties using OM will be discussed in the next section.

**Figure 6:**
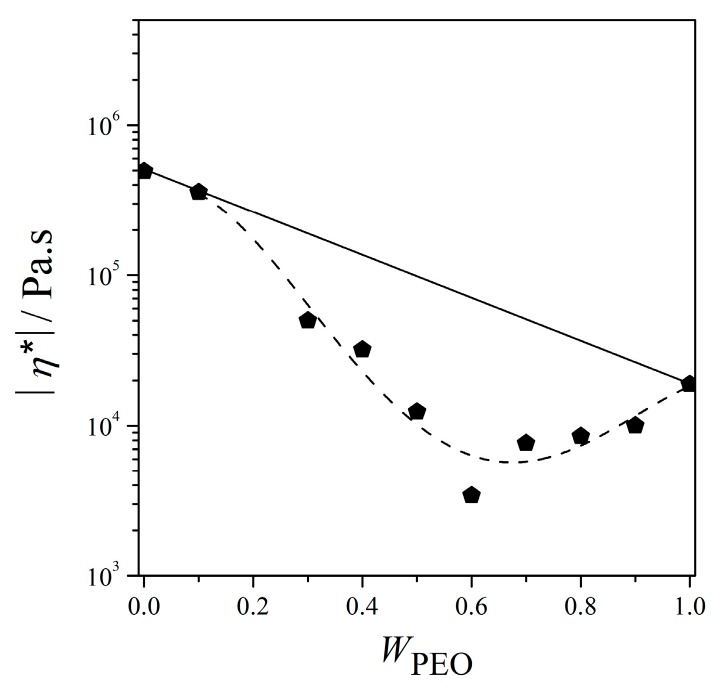
Viscosity evaluated at *f* = 0.01 Hz as a function of mass fraction of PEO at *T* = 140 °C. The solid curve represents the additive curve as in Equation (7). The dashed curve is the 4th polynomial regression curve with *R*^2^ > 0.95 for visual inspection (*cf.* text).

The qualitative evaluation for the viscous properties of the blends were done by using polynomial curve fitting using Equation (6) with *R*^2^ > 0.99 to determine the viscosity of the blends at *f* = 0.01 Hz in Figure 5. Substituting a frequency of 0.01 Hz into Equation (6), the viscosity (*η*) of the blends can be estimated as:

(6) $$\log\eta *=\eta_{0}+\eta_{1}\log f+\eta_{2}\log{\left(f\right)}^{2},$$

where coefficient $\eta_{0}$ is the zero shear-rate viscosity and coefficients $\eta_{1}$ and $\eta_{2}$ are the two-material parameters. The estimated η* values of the blends are plotted in Figure 6. The negative deviation was clearly observed for the blends in reference to the linear relationship between logη* and the composition as given in Equation (7):

(7) $$\log{\eta^{*}}_{\mathrm{PEO}+\mathrm{NR}-g-\mathrm{PMMA}}=W_{\mathrm{PEO}}\log{\eta^{*}}_{\mathrm{PEO}}+W_{\mathrm{NR}-g-\mathrm{PMMA}}\log{\eta^{*}}_{\mathrm{NR}-g-\mathrm{PMMA}},$$

where $\eta {*}_{\mathrm{PEO}}$ and $\eta {*}_{\mathrm{NR}-g-\mathrm{PMMA}}$ are the viscosity values of PEO and NR-*g*-PMMA estimated at *f* = 0.01 Hz, respectively. The $\eta {*}_{\mathrm{PEO}}$ and $\eta {*}_{\mathrm{NR}-g-\mathrm{PMMA}}$ values used in Equation (7) were 18,828 and 494,310 Pa·s, respectively, which were estimated using Equation (6).

For the blends, the viscosities were quite close to the regression curve using Equation (6). The viscosities of the blends reduced in the course of blending until up to 60 wt % of PEO, before increase with the addition of higher contents of NR-*g*-PMMA. It described that the viscosity of the blends generally decreased from both sides and reached a minimum at the blend with a roughly mass ratio of 60/40. However, the addition of *W*_PEO_ of 0.1 to NR-*g*-PMMA did not remarkably reduce the viscosity of NR-*g*-PMMA, but on the other side of blend compositions, the addition of *W*_NR-*g*-PMMA_ of 0.1 only reduced the viscosity of PEO notably. This is in agreement with $G^{\prime }$ and $G^{\prime \prime }$ as a function of frequency as in Figure 3a,b. This suggested that the blending of these two constituents generally reduced the viscosity as compared to the neat polymers and the reduction in viscosity was less when NR-*g*-PMMA was in excess. The minimum frequency of |η*| at a PEO/NR-*g*-PMMA blend composition of 60/40 may suggest a transition from a droplet-matrix phase to a co-continuous phase when NR-*g*-PMMA was added to PEO, which was also supported by the modulus behavior in Figure 3.

#### 3.2.3. Van Gurp-Palmen Analysis of the PEO/NR-*g*-PMMA Blends

**Figure 7:**
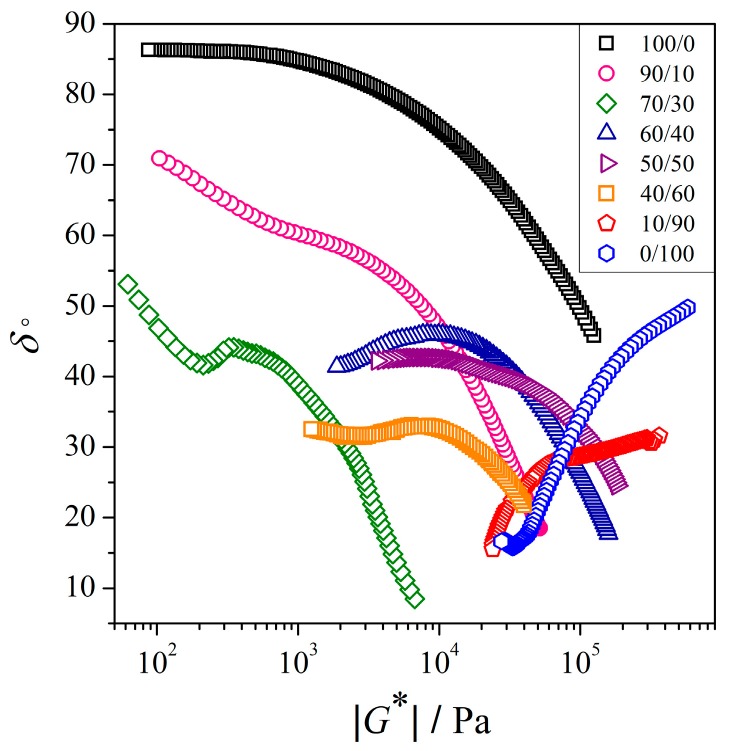
Van Gurp-Palmen plots of PEO/NR-*g*-PMMA blends at *T* = 140 °C.

The plots of phase angle (*δ*) as a function of absolute complex modulus (|G*|), also known as Van Gurp-Palmen plots, are shown in Figure 7. *δ* was the phase difference between the applied strain and the measured stress. A purely elastic material exhibited in-phase waves (*δ* = 0°), and a purely viscous material showed two out-of-phase waves (*δ* = 90°). As shown in Figure 7, the neat PEO exhibited behavior that is typical for linear polymers, where the *δ* value reached a plateau region close to 90° at lower |G*| regions, indicating the dominant viscous behavior. On the other hand, the neat NR-*g*-PMMA showed an immediate decrease in *δ* values, which implied the solid-like behavior. The Van Gurp-Palmen plot may also be considered as a complementary tool to investigate the morphology of polymer blends [18,60,61]. For a two-phase binary blends with a droplet-matrix morphology, a valley shape at the lower |G*| regions is presented [18,60,61]. In the case of co-continuous morphology, a maximum of *δ* is observed, before it descends with decreasing |G*| [60,62]. Based on Figure 7, the PEO/NR-*g*-PMMA blends with mass ratios of 90/10, 70/30, and 40/60 may imply a droplet-matrix morphology, whereas the PEO/NR-*g*-PMMA blends with mass ratios of 60/40 and 50/50 may imply a co-continuous morphology. *δ* of the blends decreased with the increasing content of NR-*g*-PMMA in the blends, which indicated a solid-like behavior. Moreover, a significant reduction in *δ* to a lower value than 45° observed for the blends with less than 60 wt % of PEO content implied strong elastic behavior. Hence, these Van Gurp-Palmen findings are in good agreement with the modulus results presented in Figure 3.

### 3.3. Morphological Studies of PEO/NR-g-PMMA Blends

**Figure 8:**
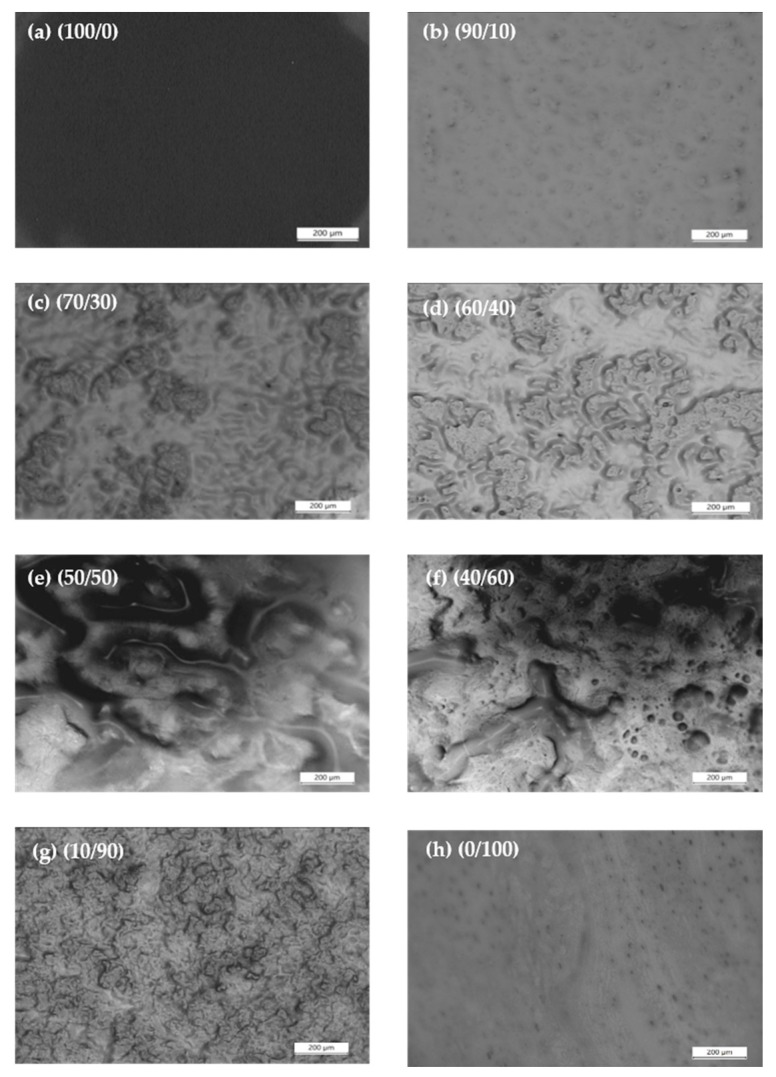
Optical microscopy images of PEO/NR-*g*-PMMA for (**a**) 100/0 (**b**) 90/10 (**c**) 70/30 (**d**) 60/40 (**e**) 50/50 (**f**) 40/60 (**g**) 10/90 and (**h**) 0/100 blends captured at *T* = 140 °C after 10 min.

The methods used for blend preparation described in Section 2.1 may provide a close thermodynamic equilibrium state for the blends. For the morphological studies of the PEO/NR-*g*-PMMA blends using OM, the samples were heated to 140 °C, and the micrographs were captured after 10 min of annealing at *T* = 140 °C. The morphologies of the blends, thus, can be assumed to be close to the equilibrium state. The micrographs of the neat PEO and its blends are shown in Figure 8. The phase separation of PEO and NR-*g*-PMMA in the melt was clearly seen in the blends. The two phases observed were either a droplet-matrix phase or a co-continuous phase. At the PEO/NR-*g*-PMMA mass ratio of 90/10, the low-viscosity PEO easily formed a matrix phase due to the minimized energy of dissipation in the terminal flow [63], as shown earlier in Figure 3a. On the other hand, the high-viscosity NR-*g*-PMMA formed a dispersed phase in the PEO matrix. To develop the same extent of NR-*g*-PMMA continuity as in the PEO/NR-*g*-PMMA blends with mass ratios of 90/10 and 80/20 (figure not shown), the mass fraction of the higher-viscosity NR-*g*-PMMA component had to be increased to maintain the connectivity of NR-*g*-PMMA phases. As observed in Figure 8d, with 40 wt % of NR-*g*-PMMA content in the blend, the NR-*g*-PMMA phases started to coalesce and became continuous. 

In immiscible binary blends, a co-continuous structure can be formed over a certain interval of volume fractions [63]. Besides, the range of co-continuity can be very narrow or wide and occurs around the phase inversion composition, *W*_PI_, which is also known as phase inversion point [63]. Furthermore, co-continuous compositions are normally found to have lower viscosities as compared to the parent polymers or the dispersed structures at higher or lower concentrations [64,65]. Similarly, for the behavior of the modulus, during the coarsening process of the co-continuous morphology, the relaxation of the dispersed phases occurred at very low frequencies and caused the $G^{\prime }$ values of the PEO/NR-*g*-PMMA blends with mass ratios of 60/40 and 70/30 to decrease [66], which were observed in Figure 3a. As observed in Figure 8d,e, co-continuous structures occurred in the PEO/NR-*g*-PMMA blends with mass ratios of 60/40 and 50/50. These observations are in agreement with the Van Gurp-Palmen plots shown in Figure 7, which indicated the co-continuous morphology occurred in the PEO/NR-*g*-PMMA blends with mass ratios of 60/40 and 50/50. The viscosity analysis in Figure 6 also agrees with the above statements, where the PEO/NR-*g*-PMMA blends with mass ratios of 60/40 and 50/50 had lower viscosity than the other blends. Besides, based on the minimum frequency of |η*| obtained by the 4th-order polynomial fitting (*cf.* Figure 6), it was suggested that phase inversion occurred for the PEO/NR-*g*-PMMA blend with a mass ratio of 60/40 when NR-*g*-PMMA was added to the PEO matrix. The micrograph of the PEO/NR-*g*-PMMA blend with a mass ratio of 60/40 (*cf.* Figure 8e) gave credence to the phase inversion prediction, where the continuous phases were both PEO and NR-*g*-PMMA.

The morphologies of PEO/NR-*g*-PMMA blends were summarized here. The droplet-matrix morphology was formed in the PEO/NR-*g*-PMMA blends with mass ratios of 90/10 and 70/30, where the droplets of NR-*g*-PMMA as a minor phase were dispersed in the PEO matrix. As the NR-*g*-PMMA content increased, the morphology showed a beginning of the continuity domain, where the NR-*g*-PMMA phase started to form a co-continuous structure in the PEO/NR-*g*-PMMA blend with a mass ratio of 60/40. As the amount of PEO decreased in the blend, this co-continuous structure of PEO broke up with some droplets coexisting with the percolated structures of PEO, as shown in Figure 8f,g. For the blends with *W*_PEO_ < 0.5, the high modulus of NR-*g*-PMMA (described in Figure 3) and *δ* lower than 45° (described in Figure 7) implied the solid-like behavior of the blends.

## 4. Conclusions

In this study, the rheological properties and morphologies of PEO/NR-*g*-PMMA blends were investigated. A transition from the liquid-like behavior of PEO to the solid-like behavior was observed, when 10 wt % of NR-*g*-PMMA was added. Based on a three-zone model of dynamic moduli, shifts from the terminal zone behavior via the plateau zone till the transition zone to the glassy state were observed upon the addition of NR-*g*-PMMA to PEO. The variation of relaxation time with blend composition may suggest that the slow relaxation time of NR-*g*-PMMA retarded the relaxation process of PEO as the amount of NR-*g*-PMMA in the blend increased. Based on the OM studies, droplet-matrix and co-continuous morphologies were observed in the PEO/NR-*g*-PMMA blends, where co-continuity was observed in the PEO/NR-*g*-PMMA blends with a composition range of 60/40–50/50. This was also supported by the Van Gurp-Palmen analysis, which suggested the droplet-matrix morphology occurred in the PEO/NR-*g*-PMMA blends with mass ratios of 90/10, 70/30, and 40/60 and the co-continuous morphology occurred in the PEO/NR-*g*-PMMA blends with mass ratios of 60/40 and 50/50 under the studied experimental conditions. Meanwhile, phase inversion was suggested to occur in the PEO/NR-*g*-PMMA blend with a mass ratio of 60/40, which was supported by the minimum viscosity and modulus analysis. 

## Figures and Tables

**Table 1:** Characteristics of poly(ethylene oxide) (PEO) and natural rubber-graft-poly(methyl methacrylate) polymer (NR-*g*-PMMA) used in this work.

Constituent	PEO	NR-*g*-PMMA
*M*_η_^a^ (g·mol^−1^)	300,000	–
*M*_w_^b^ (g·mol^−1^)	–	394,000
*M*_n_^b^ (g·mol^−1^)	–	116,000
*T*_m_^c^ (°C)	66	–
Δ*H*_ref_ (J·g^−1^)	188.3 ^e^	–
*T*_g_^d^ (°C)	−58	−66, 120
Molecular structure	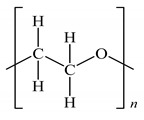	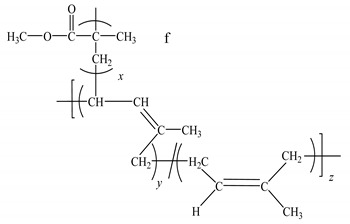
Supplier	Thermo Fisher, Pittsburgh, PA, USA	Green HPSP (M) SdnBhd, Petaling Jaya, Malaysia

^a^ Viscosity-average molar mass estimated by the supplier;

^b^ Mass-average or number-average molar mass as estimated in this work by GPC. PMMA with low dispersity was used as a standard;

^c^ Melting temperature by DSC as determined in this work;

^d^ Glass transition temperature after quench cooling by DSC as determined in this work;

^e^ Melting enthalpy adopted from Lide [40];

^f^*x* represents the mole fraction of the PMMA-graft polymer, which was equal to 0.4; *y + z* represents the mole fraction of the NR backbone, which was equal to 0.6.

**Table 2:** Power law exponents of $G^{\prime }$ and $G^{\prime \prime }$ for PEO/NR-*g*-PMMA blends at *T* = 140 °C estimated with Equation (3).

PEO/NR-*g*-PMMA Blends.	Power law exponent of $G^{\prime }$	*R* ^2^	Power law exponent of $G^{\prime \prime }$	*R* ^2^	$(f_{\mathrm{cross}}^{G^{\prime },G^{\prime \prime }})\;$(Hz)	Remark (*c.f* Figure 4a)
100/0	1.09 ± 0.02	0.997	0.91 ± 0.01	0.999	0.90 ^b^	Transition from a terminal zone to a plateau zone
	1.08 ± 0.12 ^a^	–	0.88 ± 0.08 ^a^	–	–	
90/10	1.07 ± 0.03	0.992	0.91 ± 0.02	0.995	0.50 ^b^	
70/30	0.62 ± 0.01	0.998	0.58 ± 0.01	0.999	0.11 ^b^	
60/40	0.83 ± 0.02	0.999	0.81 ± 0.01	0.999	0.080 ^b^	
50/50	0.53 ± 0.01	0.999	0.52 ± 0.01	0.999	–	Plateau zone
40/60	0.34 ± 0.03	0.999	0.33 ± 0.01	0.998	–	
10/90	0.17 ± 0.01	0.978	0.36 ± 0.01	0.999	–	
0/100	0.12 ± 0.01	0.987	0.14 ± 0.02	0.891	20 ^c^	Transition from a plateau zone to a transition zone

^a^ Values were extracted from [42];

^b^ Long-range relaxations;

^c^ Short-range relaxations.
